# Genetic diversity of *Arcobacter* isolated from bivalves of Adriatic and their interactions with *Mytilus galloprovincialis* hemocytes

**DOI:** 10.1002/mbo3.400

**Published:** 2016-09-20

**Authors:** Donatella Ottaviani, Francesco Mosca, Serena Chierichetti, Pietro Giorgio Tiscar, Francesca Leoni

**Affiliations:** ^1^ Sezione di AnconaLaboratorio Nazionale di Riferimento (LNR) Contaminazioni Batteriologiche Molluschi Bivalvi ViviIstituto Zooprofilattico Sperimentale dell'Umbria e delle MarcheAnconaItaly; ^2^ Facoltà di Medicina VeterinariaTeramoItaly

**Keywords:** *Arcobacter butzleri*, *Arcobacter cryaerophilus*, genetic diversity, hemocytes, mussels, phagocytosis

## Abstract

The human food‐borne pathogens *Arcobacter butzleri* and *A. cryaerophilus* have been frequently isolated from the intestinal tracts and fecal samples of different farm animals and, after excretion, these microorganisms can contaminate the environment, including the aquatic one. In this regard, *A. butzleri* and *A. cryaerophilus* have been detected in seawater and bivalves of coastal areas which are affected by fecal contamination. The capability of bivalve hemocytes to interact with bacteria has been proposed as the main factor inversely conditioning their persistence in the bivalve. In this study, 12 strains of *Arcobacter* spp. were isolated between January and May 2013 from bivalves of Central Adriatic Sea of Italy in order to examine their genetic diversity as well as in vitro interactions with bivalve components of the immune response, such as hemocytes. Of these, seven isolates were *A. butzleri* and five *A. cryaerophilus*, and were genetically different. All strains showed ability to induce spreading and respiratory burst of *Mytilus galloprovincialis* hemocytes. Overall, our data demonstrate the high genetic diversity of these microorganisms circulating in the marine study area. Moreover, the *Arcobacter–*bivalve interaction suggests that they do not have a potential to persist in the tissues of *M. galloprovincialis*.

## 1 Introduction

The genus *Arcobacter* has become increasingly important in recent years because species such as *A*. *butzleri* and *A. cryaerophilus* are considered emergent food‐borne enteropathogens (Collado & Figueras, 2011). *Arcobacter butzleri*, in particular, has been classified as a serious hazard to human health by the International Commission on Microbiological Specifications for Foods (ICMSF 2002) and a significant zoonotic pathogen (Cardoen et al., 2009). In Europe, *A. butzleri* was often recovered from samples of patients with diarrhea in Belgium, France, and Italy (Prouzet‐Mauleon, Labadi, Bouges, Menard, & Megraud, 2006; Vandamme et al., 1992; Vandenberg et al., 2004). Moreover, *A. butzleri* and *A. cryaerophilus* have been isolated from the intestinal tract and fecal samples of different farm animals (Van Driessche, Houf, Van Hoof, De Zutter, & Vandamme, 2003). Once they are excreted, these microorganisms can contaminate the environment, including the aquatic one (Collado, Inza, Guarro, & Figueras, 2008). In coastal areas particularly exposed to human and animal fecal pollution, *A. butzleri* and *A. cryaerophilus* can also contaminate sea water and, consequently, bivalves (Collado & Figueras, 2009; Collado, Guarro, & Figueras, 2009; Fernandez, Villanueva, Mansilla, Gonzalez, & Latif, 2015; Fernandez et al., 2001; Laishram, Rathlavath, Lekshmi, Kumar, & Nayak, 2016; Llobet‐Brossa, Roselló‐Mora, & Amann, 1998; Mottola et al., 2016; Nieva‐Echevarria, Martinez‐Malaxetxebarria, Girbau, Alonso, & Fernández‐Astorga, 2013; Romero, García‐Varela, Laclette, & Espejo, 2002; Vandieken et al., 2012). Arcobacters have been frequently reported in the marine environment and bivalves of Italy and Spain (Fera et al., 2004, 2010; Gugliandolo, Irrera, Lentini, & Maugeri, 2008; Levican, Collado, Yustes, Aguilar, & Figueras, 2014; Maugeri, Carbone, Fera, Irrera, & Gugliandolo, 2004; Maugeri, Gugliandolo, Carbone, Caccamo, & Fera, 2000). Because of their filter‐feeding activities, edible bivalves pose a high risk to human health in relation to the ability of viruses and bacteria to be entrapped in the host tissues (Potasman, Paz, & Odeh, 2002). In order to decrease the number of unwanted microorganisms to acceptable levels for human consumption, bivalve depuration and relaying in controlled waters are used worldwide (Richards, 1988). However, differences in the efficiency of bivalve depuration are known to be dependent on the species of microorganisms and their different ability to persist and multiply in the host tissues (Maalouf et al., 2011; Marino, Crisafi, Maugeri, Nostro, & Alonzo, 1999; Perkins, Haven, Morales‐Alamo, & Rhodes, 1980; Prieur, Mevel, Nicolas, Plusquellec, & Vigneulle, 1990; Pruzzo, Gallo, & Canesi, 2005; Richards, 1988). In marine bivalves, the cell‐mediated immunity constitutes the main internal defense in response to invading organisms and the circulating hemocytes represent the phagocytic cells which are able to recognize, engulf, and destroy the foreign particles (Bachère et al., 1995). Bivalve hemocytes can kill microbes through phagocytosis and various cytotoxic reactions, such as the release of lysosomal enzymes and antimicrobial peptides (Canesi, Gallo, Gavioli, & Pruzzo, 2002; Pruzzo et al., 2005). Ability of bacteria to escape hemocytes killing activity directly affects their persistence in bivalve tissues and, consequently, their resistance to depuration (Canesi, Gallo, Gavioli, & Pruzzo, 2002; Canesi, Pruzzo, Tarsi, & Gallo, 2001; Harris‐Young, Tamplin, Mason, Aldrich, & Jackson, 1995). Moreover, the persistence of microorganisms in bivalves can be species or strain dependent (De Abreu Corrìa et al., 2007; Maalouf et al., 2011; Morrison et al., 2011). This is not surprising considering that phagocytosis requires specific surface interactions with bacteria/bivalve (Canesi et al., 2002). Phagocytic efficiency of bivalve hemocytes toward foreign particles can be assessed by studying the hemocyte ability to spread toward them (Malagoli & Ottaviani, 2004; Mosca, Narcisi, Cargini, Calzetta, & Tiscar, 2011; Mosca, Lanni et al., 2013; Mosca, Narcisi, et al., 2013) and degrade these by producing reactive oxygen species (ROS). This latter activity is defined respiratory burst (Gunderson & Seifert, 2015; Ordàs, Novoa, & Figueras, 2000; Versleijen et al., 2008). To our knowledge, only one previous work studied the dynamic of interaction of *A. butzleri* and bivalves showing that the type strain *A. butzleri* LMG 10828 T did not persist in *Mytilus galloprovincialis* (Ottaviani, Chierichetti et al., 2013). In order to acquire preliminary information about potential persistence of *Arcobacter* spp. in host tissues, it is important to investigate the circulation of human pathogenic species in different marine environments and possible inter‐ and intraspecies differences in the interaction with the bivalves that are more commonly harvested in those areas. Despite this, reports on the genetic characterization of arcobacters isolated from the marine environment and pathogen–bivalve immunologic interaction are scarce (Collado, Jara, Vásquez, & Telsaint, 2014; Levican et al., 2014; Ottaviani, Chierichetti et al., 2013). In this study *Arcobacter* strains, isolated from bivalves collected from a restricted harvesting area of the Central Adriatic Sea of Italy, were molecularly identified and typed by enterobacterial repetitive intergenic consensus PCR (ERIC‐PCR) analysis. Moreover, spreading activity and respiratory burst induced by these isolates on hemocytes of *M. galloprovincialis*, the most common bivalve harvested in the Central Adriatic Sea, were investigated.

## 2 Material and Methods

### 2.1 Sample collection and processing

**Figure 1:**
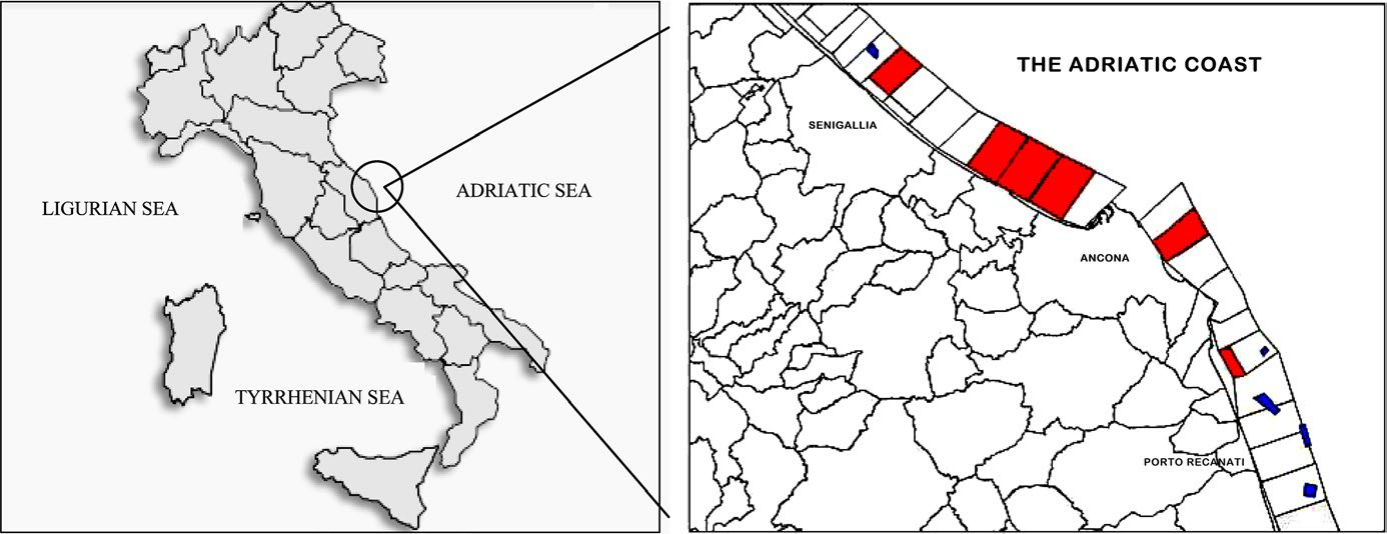
Geographical location of sampling areas in the Central Adriatic coast of Italy (Marches Region): blue and red sites correspond to *Mytilus galloprovincialis* and *Chamelea gallina* sampling areas, respectively

Forty samples of bivalves were collected between January and May 2013 from multisites of an authorized harvesting Class B area of the Central Adriatic Sea (Fig. 1). Bivalve mollusks from these areas must not exceed 4600 MPN *Escherichia coli* per 100 g and must be depurated before human consumption (Regulation EC No. 854/2004). In particular, at each sampling performed weekly, one sample of *M. galloprovincialis* and one of *Chamelea gallina* were collected, for a total of 20 samples for each of the two species of bivalves. Standardized methods were followed for the preparation of shellfish samples (ISO 6887‐3, 2003; Ottaviani et al., 2015) and to detect *Arcobacter* (Collado, Cleenwerck, Van Trappen, De Vos, & Figueras, 2009). Briefly, 10 g of flesh and liquor were homogenized with 90 ml (1:10, wt/vol) of *Arcobacter* broth supplemented with cefoperazone, amphotericin B, and teicoplanin (*Arcobacter*‐CAT broth). After an incubation at 30°C for 48 hr in aerobic conditions, 0.2 ml of the broth were inoculated by passive filtration with Millipore filters (0.45 μm, 47 mm) onto blood agar plates (trypticase soy agar supplemented with 5% sheep blood) and incubated for 48–72 hr at 30°C under aerobic conditions.

### 2.2 Identification and molecular characterization of *Arcobacter* isolates

Presumptive colonies biochemically identified as *Arcobacter* spp. (Collado et al., 2009) were tested by PCR for the *rpoB* gene of *Arcobacter* spp. (Collado et al., 2009) with primers from Korczak et al. (2006). In brief, 4–5 colonies from each isolate were suspended in 500 μl of sterile distilled water and denatured at 95°C for 10 min, then centrifuged at 13.000 rpm for 1 min. The supernatant was recovered and tested immediately or stored at −20°C. A fragment of the *rpoB* gene was amplified in a final volume of 50 μl containing 1× PCR buffer (GoTaq Promega), 0.2 mmol/L of each dNTP, 0.5 μmol/L of each primer (primers CamrpoB‐L and RpoB‐R from Korczak et al., 2006), 2.5 mmol/L of MgCl_2_ (GoTaq Promega), 0.05 U/μl of Taqpol (Go Taq Promega), and 5 μl of lysate. PCR conditions were as follows: 30 cycles at 94°C for 30 s, 54°C for 30 s, and 72°C for 30 s, and a final elongation at 72°C for 5 min. PCR products (10 μl) were checked for the presence of the *rpoB* fragment by horizontal electrophoresis in 1% agarose gel containing 0.5% of ethidium bromide. Species identification of *Arcobacter* isolates was performed by sequencing analysis of *rpoB* gene fragments. In brief, PCR products for sequencing analysis were purified with the High Pure PCR Product Purification kit (Roche Diagnostics). Sequencing analysis was performed in both directions with a ABI Prism^®^ BigDye^®^ Terminator v1.1 Cycle Sequencing kit (Life Technologies), according to the manufacturer's instructions, using *rpoB* primers and an automated capillary sequencer ABI Prism^®^ 310 Genetic Analyzer (Applied Biosystems). The genetic diversity among *Arcobacter* isolates was assessed using ERIC‐PCR method standardized by Houf, De Zutter, Van Hoof, and Vandamme (2002). In brief, the 50‐μl mixture contained 5 or 10 μl PCR buffer (Invitrogen, Carlsbad, CA), 5 U of *Taq* polymerase (Invitrogen), deoxynucleoside triphosphates at a final concentration of 0.2 mmol/L each (Invitrogen), 1.3 μl of 50 mmol/L MgCl_2_ (Invitrogen), 25 pmol of each of the primers (ERIC‐1R, 5′–ATGTAAGCTCCTGGGGATTCAC–3′; and ERIC‐2, 5′–AAGTAA GTGACTGGGGTGAGCG–3′), 25 pg of DNA template, and Milli‐Q water. The PCR consisted of an initial denaturing at 94°C for 5 min followed by 40 cycles of 94°C for 1 min, 25°C for 1 min, and 72°C for 2 min, with a final extension at 72°C for 5 min. *Arcobacter butzleri* LMG 10828^T^ was used as the reference strain in all molecular reactions.

### 2.3 Experimental animals and hemolymph collection for phagocytosis assays

Each phagocytosis assay was performed on hemolymph pooled from 10 organisms of *M. galloprovincialis* (40–50 mm shell length) harvested from the same coastal area from which bivalves sampling was carried out. Moreover, each phagocytosis assay was repeated five times on five different lots of mussels, monthly sampled from January to May 2013. The organisms were maintained in a closed recirculating system, equipped with a filtering apparatus and air supply. A multiparameter probe (mod. YSI 556; Technosea) was used to monitor the temperature (mean 18.1 ± 0.6°C), salinity (mean 33.1 ± 0.2‰), and dissolved oxygen (DO) (mean 6.8 ± 0.3 mg/L). The mussels were acclimated for 48 hr at least before the experiment and no feed was administered. The hemolymph was extracted from the posterior adductor muscle, it was pooled and the cell concentration was adjusted to 1 × 10^6^/ml in ice‐cold artificial sterile seawater (ASS). The pool was then subdivided into two aliquots for phagocytosis assays. For both assays, cell wall of *Saccharomyces cerevisiae* (Zymosan A; Sigma‐Aldrich) was used as the gold standard for its very high ability to stimulate phagocytosis (Malagoli & Ottaviani, 2004; Mosca 2013a,b; Ordàs et al., 2000). Moreover, not stimulated hemolymph represented the basal level. For the experiments on hemocytes, the *Arcobacter* strains, grown in tryptone soy broth (Oxoid) at 30°C for 48 hr, were centrifuged at 5000*g* for 20 min at 4°C. Pellet was resuspended in phosphate buffer saline (PBS, 10% w/v) and adjusted to the concentration of 5 × 10^9 ^CFU/ml after optical measurement (5 × 10^8^ CFU/ml gave approximately 0.5 OD600 nm).

### 2.4 Spreading activity

The spreading activity of hemocytes was investigated, evaluating changes in hemocyte shape from a rounded (inactive) to an ameboid (active) form. In particular was measured the shape factor (SF), a pure number quantifying the degree to which a cell deviates from circularity, by using a video imaging system integrated into a computerized analyzer of cells in suspension (Cell Viability Analyzer Beckman Coulter) (Malagoli & Ottaviani, 2004). An aliquot of each hemolymph sample was incubated for 30 min with bacterial suspension, corresponding to bacteria to hemocytes ratio of 80:1 (Mosca et al., 2011). Ninety μl of the mixture was then placed on a microscope slide within a chamber delimited by a vaseline ring. A coverslip was placed over the slide in order to partially cover the chamber and, following hemocyte adhesion (time 0), 10 μl of 10 1 μmol/L N‐formyl‐Meth‐Leu‐Phe (fMLP) was added to the uncovered part of the chamber. The digital image of the hemocytes was acquired after 20 min and the parameters for SF evaluation, that is, perimeter length and area, were automatically obtained by electronically tracing the edges of the cell. The assay was assayed in parallel with Zymosan A using a yeast to hemocytes ratio of 80:1 and untreated hemolymph (Mosca et al., 2011). Global results from five independent experiments for each test and control group were expressed as SF mean values ± standard deviation (*SD*).

### 2.5 Hemocyte respiratory burst

Luminol‐enhanced chemiluminescence (CL) assay has been widely used to measure the production of ROS in bivalve hemocytes (Gunderson & Seifert, 2015; Mosca et al., 2011; Mosca, Lanni et al., 2013; Mosca, Narcisi, et al., 2013; Ordàs et al., 2000; Versleijen et al., 2008). The measurement of the luminescence intensity (*y*‐axis) versus time (*x*‐axis) generates a positive polynomial curve. So, the results can be reported as integral value of the area under curve (AUC) (Gunderson & Seifert, 2015; Versleijen et al., 2008). For CL assay, hemolymph samples were placed in triplicate in 96‐well microplates. Following 15 min incubation with Luminol (Sigma Aldrich) at 1 mmol/L, the hemocytes were stimulated to phagocytosis with *Arcobacter* suspensions (bacteria to hemocytes ratio of 80:1) and Zymosan A (zymosan to hemocytes ratio of 80:1). The chemiluminescence intensity by microplate reader (Sinergy H1; Bio‐Tek) was detected for 60 min by plotting the curve of the counts per min and then calculating the AUC by integration (Gunderson & Seifert, 2015; Versleijen et al., 2008). Global results from five independent experiments for each test and control group were expressed as AUC mean values ± standard deviation (*SD*).

### 2.6 Statistical analysis

For the analysis of ERIC‐PCR electrophoresis products, dendrograms were constructed by the use of the Dice coefficient (Dice, 1945) and the unweighted‐pair group method using arithmetic averages (software Treeconw version: 1.3b, Copyright © Yves Van de Peer University of Konstanz). Genetic relatedness between the PCR electrophoresis products of the strains was interpreted according to the method of Tenover et al. (1995). Thus, isolates were designated as indistinguishable, closely related; possibly related, and different with 0, 1–3, 4–6, >7 band differences, respectively (Tenover et al., 1995). Clusters were defined on the basis of the 80% similarity cutoff (Ottaviani, Leoni et al., 2013). In the phagocytosis assays, the statistical significance of differences between means was determined by one‐way ANOVA for paired samples (each strain against each other and each control). The level for accepted significance was *p* < .01.

## 3 Results

### 3.1 Prevalence of *Arcobacter* from bivalves

Of the 40 samples, 12 (30%), collected from 11 different sites, were positive for the presence of presumptive *Arcobacter* spp. (Table 1). Seven strains (58%) were isolated from *C. gallina* and 5 (42%) from *M. galloprovincialis* (Table 1). Six strains (50%) were isolated in April, 2 (17%) in each month of January and March, and 1 (8%) in each month of February and May (Table 1). However, the number of strains is too low to assess whether differences of prevalence are significant.

**Table 1:** *Arcobacter* strains analyzed in this study

*Arcobacter* strains	Period of isolation	Source	Species	Collection site
3132	09 January 2013	*Mytilus galloprovincialis*	*A. cryaerophilus*	Site 1
3133	17 January 2013	*Mytilus galloprovincialis*	*A. butzleri*	Site 2
7505/1	18 February 2013	*Mytilus galloprovincialis*	*A. cryaerophilus*	Site 3
12399/2	20 March 2013	*Mytilus galloprovincialis*	*A. cryaerophilus*	Site 4
12399/3	20 March 2013	*Mytilus galloprovincialis*	*A. cryaerophilus*	Site 5
14292/2	04 April 2013	*Chamelea gallina*	*A. butzleri*	Site 6
14292/5	04 April 2013	*Chamelea gallina*	*A. butzleri*	Site 7
15366/3	10 April 2013	*Chamelea gallina*	*A. butzleri*	Site 8
15366/4	10 April 2013	*Chamelea gallina*	*A. butzleri*	Site 9
15366/6	10 April 2013	*Chamelea gallina*	*A. butzleri*	Site 10
15366/7	10 April 2013	*Chamelea gallina*	*A. butzleri*	Site 11
19042/2	06 May 2013	*Chamelea gallina*	*A. cryaerophilus*	Site 11

### 3.2 Molecular characterization of the strains

**Figure 2:**
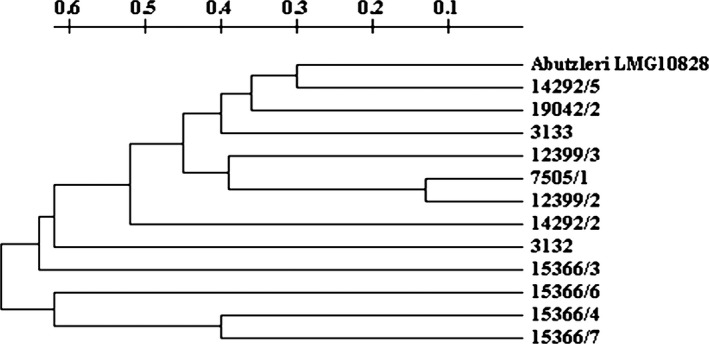
Dendrogram of *Arcobacter* isolates. In the scale, 0.0 corresponds to 100% similarity

The results of *Arcobacter* molecular identification are summarized in Table 1. All isolates, presumptively identified as *Arcobacter* spp., were also confirmed to belong to *Arcobacter* genus by *rpoB* PCR analysis. Moreover, of 12 isolates, 7 (58%) and 5 (41%) were identified as *A. butzleri* and *A. cryaerophilus*, respectively, by sequencing of *rpoB* gene. Of seven *A. butzleri* strains, 1 (14%) was isolated from *M. galloprovincialis* and 6 (86%) from *C. gallina*. Of five *A. cryaerophilus* strains, 4 (80%) were isolated from *M. galloprovincialis* and 1 (20%) from *C. gallina*. Of seven *A. butzleri* strains, 6 (86%) were isolated in April and 1 (24%) in January. Of five *A. cryaerophilus* strains, 2 (40%) were isolated in March and 1 (20%) in each month of January, February, and May. However, number of strains is too low to assess whether differences in the source and seasonality of isolation are significant. The molecular characterization of *Arcobacter* isolates performed by ERIC‐PCR revealed 12 different patterns by Tenover criteria. *Arcobacter cryaerophilus* strains 7505/1 and 12399/2, both isolated from *M. galloprovincialis* in February and March, respectively, belonged to the same cluster with a similarity between 80% and 90% (Table 1; Fig. 2). The other 10 strains belonged to different clusters with a similarity lower than 80%. Of these, *A. butzleri* 14292/5 strain and *A*. *cryaerophilus* strain 19042/2 isolated from *C. gallina* in April and May, respectively, had a similarity between 60% and 70% (Table 1; Fig. 2). All the others had a similarity equal or lower than 60% (Table 1; Fig. 2).

### 3.3 Immune response

All data of spreading activity are summarized in Table 2. Respect to the basal level, *Arcobacter* isolates and *A. butzleri* reference strain induced significant spreading activity of hemocytes ranging the SF values from 0.605 to 0.696. No significant difference of SF values was detected among strains. Moreover, SF value of Zymosan A was not significantly different from those of *Arcobacter* strains. All data of respiratory burst are summarized in Table 2. Compared to the basal level, *Arcobacter* isolates and *A. butzleri* reference strain induced significant CL responses with AUC values ranged between 3.2 and 3.7. Moreover, no significant intra‐ and interspecies differences were observed among strains. Nevertheless, the stimulation with Zymosan A induced AUC value significantly higher compared to those induced by *Arcobacter* strains (Table 2).

**Table 2:** Phagocytosis assays

	Spreading activityShape factor value (mean ± SD; *N* = 5)	Respiratory burstArea under the curve value (mean ± SD; *N* = 5)
Basal level	0.859 ± 0.01	0.9 ± 0.10
Zymosan A	0.638 ± 0.01	9.8 ± 0.80
*Arcobacter cryaerophilus* 3132	0.679 ± 0.01	3.5 ± 0.05
*Arcobacter cryaerophilus* 7505/1	0.693 ± 0.01	3.2 ± 0.06
*Arcobacter cryaerophilus* 12399/2	0.665 ± 0.01	3.5 ± 0.09
*Arcobacter cryaerophilus* 12399/3	0.605 ± 0.03	3.5 ± 0.2
*Arcobacter cryaerophilus* 19042/2	0.694 ± 0.02	3.4 ± 0.1
*Arcobacter butzleri* 3133	0.690 ± 0.01	3.4 ± 0.04
*Arcobacter butzleri* 142922	0.670 ± 0.01	3.5 ± 0.2
*Arcobacter butzleri* 142925	0.690 ± 0.01	3.2 ± 0.18
*Arcobacter butzleri* 153663	0.608 ± 0.03	3.3 ± 0.08
*Arcobacter butzleri* 153664	0.684 ± 0.02	3.5 ± 0.17
*Arcobacter butzleri* 153666	0.691 ± 0.02	3.7 ± 0.20
*Arcobacter butzleri* 153667	0.689 ± 0.02	3.4 ± 0.25
*Arcobacter butzleri* LMG 10828^T^	0.696 ± 0.01	3.2 ± 0.01

## 4 Discussion

*Arcobacter* species have been frequently isolated from seawater of the Mediterranean area (Fera et al., 2004, 2010; Gugliandolo et al., 2008; Maugeri et al., 2004), where they may survive for a long time (Collado et al., 2008). These results suggest that the marine environment, thus, the marine organisms used as a food, particularly bivalves, may represent a potential reservoir of *Arcobacter* for infection (Collado & Figueras, 2011). In agreement with previous studies carried out in different geographical areas, this study confirms that potentially pathogenic arcobacters are frequently found in bivalve samples and *A. butzleri* is the most prevalent species (Collado et al., 2009, 2014; Levican et al., 2014; Mottola et al., 2016; Nieva‐Echevarria et al., 2013). Previous studies in Spanish marine areas reported *A. butzleri* and *A. cryaerophilus* as the most prevalent species isolated from mussels and clams, respectively (Collado et al., 2009), while, with respect to the seasonality, *A. butzleri* predominated from June to October and *A. cryaerophilus* from January to May (Levican et al., 2014). In this study, in the monitored Adriatic Coast of Central Italy, *A. butzleri* was more frequently isolated in clams than in mussels and *A. cryaerophilus* more in mussels rather than in clams. Moreover, *A. butzleri* and *A. cryaerophilus* were isolated throughout the period of the study, from January to May. This study period covers even the coldest months of the year for this geographical area, that is, January–March when water temperatures ranges, usually, between 10 and 14°C. Our ERIC‐PCR results demonstrated that different strains of *A. butzleri* and *A. cryaerophilus* circulated in the restricted marine environment object of the study in January–May 2013. This high variability of *Arcobacter* strains circulation is in agreement with recent studies from Chile and Spain (Collado et al., 2014; Levican et al., 2014). Unfortunately, the short period of investigation allowed us to isolate a number of strains too limited to make any statistical evaluation of these data. For this reason, in a future investigation, we intend to extend the period of study in order to assess if in this marine ecosystem differences of isolation in respect of source and seasonality may exist and whether particular strains can be permanently present. The capability of bacteria to avoid the immunological reaction of hemocytes has been demonstrated as the main factor directly conditioning their potential to persist in the bivalves (Balbi et al., 2013; Canesi et al., 2001, 2002; Parisi et al., 2008; Pruzzo et al., 2005). Therefore, the elucidation of the interactions between pathogenic bacteria and hemocytes is important to explain their clearance in bivalve tissues and to predict the consequent efficiency of their elimination by depuration strategies (Pruzzo et al., 2005). The functional approach used in this study to evaluate pathogen–bivalve interactions demonstrates that all *A. butzleri* and *A. cryaerophilus* strains, including *A. butzleri* type strain, induced phagocytic answers of hemocytes significantly higher with respect to the basal level and spreading activity not significantly different from that of Zymosan. Zymosan is a highly concentrated immunogenic compound represented by the cell wall of *Saccharomyces cerevisiae* and, for this, it is usually used as the gold standard in phagocytosis assays. On the contrary, our *Arcobacter* strains were assayed without any treatment that could concentrate their antigenic components. This could explain the higher respiratory burst induced by Zymosan rather than that induced by strains. These results are consistent with bioaccumulation experiments of *A. butzleri* LMG 10828 in *M. galloprovincialis* that demonstrated how *Arcobacter* type strain was quickly removed from the host tissues (Ottaviani, Chierichetti et al., 2013). To the light of these findings it can be assumed for these *Arcobacter* strains a rapid clearance from the *M. galloprovincialis* tissues. In future investigations, to fully elucidate how bacteria–bivalve interactions develop and persist in natural condition, it is our intention also to consider the environment in which these interactions occur and to evaluate its influence on both the expression of bacterial cell properties and bivalve health status. However, to our knowledge, this study, even with these limitations, represent the first that tested, at the laboratory scale, the *Arcobacter–*mussel interactions by studying the phagocytic capability of *M. galloprovincialis* hemocytes toward *A. butzleri* and *A. cryaerophilus* strains, thus providing preliminary information to predict the efficiency of their elimination by host tissues and the consequent impact on human health.

## 5 Conclusions

Different strains of human pathogenic *A. butzleri* and *A. crioaerophilus* were found in *M. galloprovincialis* and *C. gallina* harvested from a restricted marine environment of the Central Adriatic Sea of Italy between January and May 2013. However, the interaction of these *Arcobacter* strains with *M. galloprovincialis* hemocytes suggests that they do not have a potential to persist in mussels tissues and therefore they could be efficiently removed with the conventional purification practices.

## Funding Information

This work was funded by a research project (IZSUM 10/2011) from the Italian Ministry of Health.

## Conflict of Interest

None declared.
